# GP coding behaviour for non-specific clinical presentations: a pilot study

**DOI:** 10.3399/bjgpopen20X101050

**Published:** 2020-07-08

**Authors:** John SP Tulloch, Mike BJ Beadsworth, Roberto Vivancos, Alan D Radford, Jenny C Warner, Rob M Christley

**Affiliations:** 1 National Institute for Health Research Health Protection Research Unit (NIHR HPRU) in Emerging and Zoonotic Infections, University of Liverpool, Liverpool, UK; 2 Public Health England, Liverpool, UK; 3 Tropical and Infectious Disease Unit, Royal Liverpool University Hospital, Liverpool, UK; 4 NIHR HPRU in Emerging and Zoonotic Infections, Liverpool School of Tropical Medicine, Liverpool, UK; 5 NIHR HPRU in Emerging and Zoonotic Infections, Public Health England, Porton Down, UK; 6 Institute of Infection and Global Health, University of Liverpool, Liverpool, UK; 7 NIHR HPRU in Emerging and Zoonotic Infections, Public Health England, Liverpool, UK; 8 Rare and Imported Pathogens Laboratory, Public Health England, Porton Down, UK

**Keywords:** primary health care, general practice, clinical coding, Lyme disease

## Abstract

**Background:**

Clinical coding is an integral part of primary care. Disease incidence studies based on primary care electronic health records (EHRs) rely on the accuracy of these codes. Current code validation methods are not appropriate for non-specific conditions and provide limited information about GPs' decision-making behaviour around coding. Qualitative methods could offer insight into decision-making behaviour around coding of patients with non-specific conditions.

**Aim:**

To investigate the decision-making behaviour of GPs when applying Read codes to non-specific clinical presentations, using Lyme disease as a case example.

**Design & setting:**

A pilot study was undertaken, involving masked semi-structured interviews of eight GPs in the North West of England.

**Method:**

Semi-structured interviews were carried out based on 11 clinical cases representative of Lyme disease presentations. Discrete answers were described descriptively. Interview transcripts were analysed using a thematic approach.

**Results:**

Themes underpinning GPs’ coding behaviour included: GP personal and professional experience; clinical evidence; diagnostic uncertainty; professional integrity and defensive practice; and patient-sourced health information and beliefs. GPs placed Lyme disease on their differential diagnosis list for five cases; in only two cases would GPs select a Lyme disease related Read code.

**Conclusion:**

GPs were reluctant to code with specific diagnostic Read codes when they were presented with patients with vague or unfamiliar symptomology. This masked questionnaire methodology offers a new approach to validate incidence figures, based on Read codes of non-specific conditions. The reluctance to code poses many problems for primary care EHRs research. Further research is needed to understand what drives GPs’ coding behaviour.

## How this fits in

Research based on primary care EHRs relies on Read code validation; this is challenging when the clinical presentation is equivocal. Primary care clinicians are reluctant to code with specific Read codes when presented with patients with vague or unfamiliar symptoms. Qualitative methodologies offer alternative techniques to validate the use of Read codes to describe disease incidence in primary care research.

## Introduction

Read codes, currently being replaced by SNOMED CT codes in the UK, are a hierarchical standard terminology system that encode clinical, diagnostic, and therapeutic patient information.^1^ They enable data entry of patient care information during or after a primary care consultation. This information is stored within a patient’s EHR. Their primary purpose is to digitally record summary clinical and administrative data in primary care, and were never intended for secondary analysis in epidemiological research. They have become key elements of general practice research via primary care databases, such as the Clinical Practice Research Datalink (CPRD) (https://www.cprd.com) and The Health Improvement Network (THIN).^2^ Read code usage in research has limitations in terms of: data quality; validation of codes; incomplete data capture; differences between EHR systems; variability in usage between clinicians and practices; and varied coverage across the country.^3,4^ These issues are well acknowledged but, as yet, there is no methodology to overcome them, thus all research based on Read codes must be interpreted appropriately. Despite the widespread use of Read codes, the decision-making behaviour of GPs regarding the selection of Read codes is poorly understood.^4^

Acknowledged barriers to coding include: the perception that coded data is unimportant; the difficulty of their use for complex clinical presentations; the difficulty in creating a high quality clinical record alongside a patient-centred consultation; and targets and incentives distorting coding behaviour.^4^ Many clinicians claim not to see a direct patient benefit to coding their records.^4–6^ In addition, if an EHR receives a ‘definitive diagnosis’, via a Read code, it may be harmful to the patient and damage the patient–clinician relationship.^6,7^

To interpret any research findings describing disease incidence, Read code validation studies are needed, and while several methodologies are described, each has limitations.^8,9^ Comparing incidence figures to a national database requires a reliable database, making validation of a disease or condition that either does not have a national clinical registry, or is likely to only present in primary care, challenging.^10,11^ Independent validation of health records can be performed by research teams and has been described as the most robust method, but costs around 70 GBP per record.^8^ Owing to the high expense and ethical challenge of requesting clinical notes, most studies rely solely on questionnaires,^8^ where GPs are asked how cases were confirmed. This can be through hospital confirmation letters, clinical notes, hospital records, EHRs, or laboratory reports.^8,9,12–14^ None of these methodologies provide insight into clinicians’ coding and decision-making behaviour.

The largest limitations of these methodologies are that they are not applicable to conditions with a broad or vague case definition, or those that do not require laboratory or hospital diagnostics. One such condition is Lyme disease.^15^ It is caused by a spirochaetal infection (*Borrelia burgdorferi*) spread by the bite of *Ixodes spp* of ticks,^16^ and is the most common vector-borne disease in Europe.^17^ The disease commonly presents with an erythema migrans rash and associated fever.^15^ The laboratory-confirmed incidence in the UK is 2.64 cases per 100 000 in 2017.^18^ The National Institute for Health and Care Excellence (NICE) states that cases should be managed in primary care without laboratory testing performed or hospital referral unless the case is more complicated.^15^ As a result, it is not possible to validate and interpret Lyme disease-coded primary care EHR incidence figures using current methodologies.

To interpret incidence figures based on non-specific clinical conditions using Read codes in primary care, it is vital that a new research methodology validating Read codes is developed. This cannot be constructed until the coding behaviour of GPs presented with such conditions is understood. The aim of this pilot study was to understand what shapes GP coding practices for non-specific clinical presentations, and what Read codes would be chosen for these presentations.

## Method

Masked semi-structured interviews were used to enable both the collection of discrete answers to set questions and for GP participants to expand discursively on the answers. This research was a pilot study and participant recruitment were implemented through the National Institute for Health Research’s Clinical Research Network (North West Coast). A theoretical sampling technique was used in which participant recruitment continued until the point at which the authors believed data saturation had been reached.^19,20^ Recruited GPs were informed they were taking part in a study entitled ’Understanding the decision-making process of general practitioners (GPs) presented with non-specific conditions’, thereby masking them to the study’s focus on Lyme disease. The answers generated should reflect GPs' coding practices and case management strategy closer to a real clinical setting, and would not be biased by the focus on Lyme disease.

Interviews occurred at the GPs’ practices. After basic demographic information ws collected, participants were presented, in turn, with 11 clinical cases. To reduce the impact of cognitive bias (recollection of previous cases in the interview could impact their answers to the current case), the cases were presented in a random order. Clinical cases were based on real cases and were constructed using current clinical case definitions^15,21^ and thorough discussions with infectious disease consultants at the Royal Liverpool University Hospital (see Table 1 and Supplementary Appendix S1). Most cases had vague symptomology that could have easily been attributed to a variety of causes other than Lyme disease. The GPs were told that all the patients were otherwise healthy.

For each case, a series of questions were asked by the interviewer (as outlined in Supplementary Appendix S1) regarding the GPs’ differential diagnoses, how they would code their patient (using Read codes), and decisions regarding diagnostics, prescriptions, and referrals. If the participant asked for more information about a specific case, the description contained within the interview manuscript was repeated. The GPs were encouraged to discuss how they reached their decisions. The answers to set questions were analysed descriptively. On completion of the clinical cases, the GPs were unmasked and were provided with resources about Lyme disease.

The interviews were recorded, transcribed, and thematically analysed to explore coding behaviour.^22^ The transcriptions were read multiple times to allow thorough immersion in the data. The transcripts were coded using an inductive approach, allowing thematic codes to emerge from the data rather than from pre-planned codes. The resultant codes were reread and combined into a set of themes. The themes were checked with the coded extract and the main corpus. To ensure reliability and repeatability of the results, themes were refined with members of the research team until a consensus was reached. Identified themes were organised and quotations selected to highlight the main themes. Interpretation of the themes occurred to theorise the explanatory causes behind them.

## Results

Ten GPs in the North West of England indicated that they were willing to participate. Eight GPs attended their interviews; it is unknown why two did not attend. Analysis suggested that sufficient data saturation was reached for the purposes of this study^19,20^ and further recruitment was not required. The demographics of the GPs interviewed are summarised in Table 2. The mean interview length was 43 minutes (range 20–60 minutes). Owing to time constraints in the practices and the amount of time that each GP discussed the cases, four GPs completed all the cases, three GPs completed 10 cases, and one GP completed two cases. The descriptive analysis of GP responses to the clinical cases are summarised in Table 3. Out of the 11 cases, five cases contained Lyme disease on their differential diagnosis list, and in only two cases would a GP select a Lyme disease related Read code. In most cases the differential diagnosis list differed from the list of Read codes selected.

**Table 1.** A summary of clinical cases presented to GPs (for full details see Supplementary Appendix S1)

Case	Further details
1: Classic erythema migrans (EM) rash	Initial presentation of EM. A photo used by multiple organisations, so GPs likely to be familiar with. Then patient revealed to have been bitten by a tick
2: Borrelial lymphocytoma	Borrelial lymphocytoma of the ear lobe
3: Acrodermatitis chronica atrophicans (ACA)	With associated peripheral neuropathy
4: Bell’s palsy	Later revealed to have insect bite on scalp
5: Recurrent synovitis of knees	None
6: Multiple EM rashes	Patient had been walking in Dartmoor
7: Heart rhythm abnormalities	None
8: Fatigue, post-exertional malaise, anxiety, headaches, and memory issues	Later patient reveals an international lab report saying she has Lyme disease, and she demands long-term antibiotics
9: Fatigue, arthralgia, poor ability to concentrate, myalgia, mood swings	None
10: Non-engorged tick attached to scalp	None
11: Poor fine motor movements, rash 2 months previously at scout camp. This had been treated with erythromycin.	None

**Table 2.** The demographics of eight GPs in the North West of England, who were interviewed to understand clinical-coding behaviour

**Demographics, *n* =** **8**	**Mean (range**)
Sex, male, *n*	5
Mean age, years	46 (38–53)
Mean time in clinical practice, years	16 (10–26)
**Location of practice** **,** ***n***LancashireMerseysideCheshire	521
Time taken to complete interview, minutes	43 (20–60)
**I** **nterview cases completed** **,** ***n***All 11 cases10 cases2 cases	431

**Table 3.** Summary of key results of a masked case-study questionnaire about coding and diagnosis regarding Lyme disease. If the case had split sections, the cells in the table are similarly split

**Case (number of GPs completing it**)	**Top three differential diagnosis, where *n* >2, and any mention of a Lyme disease diagnoses (*number of interviews given where mentioned*)**	**Top three Read codes, where *n* >2, and any Lyme disease Read codes ***(number of interviews given where mentioned)*****	**Participant would perform further diagnostics, *n***	**Participant would prescribe drugs, *n***	**Participant would refer the case, *n***
1: Classic erythema migrans (EM) rash (*n* = 7)	Lyme diseaseBite(*10 differentials in total*)	52	RashLyme diseaseEMSuspected Lyme disease(*5 Read codes in total*)	3311	5	5	5
	Lyme disease(*3 differentials in total*)	6					
2: Borrelial lymphocytoma (*n* = 7)	No differential was mentioned more than once.(*13 differentials in total*)		Ear swelling(*5 Read codes in total, one GP wouldn’t code the case*)	2	2	3	4
3: Acrodermatitis chronica atrophicans (*n* = 8)	Alcohol-related issueVitamin B12 deficiency(*19 differentials in total*)	43	Peripheral neuropathySensory loss(*4 Read codes in total, one GP wouldn’t code the case*)	32	8	2	4
4: Bell’s palsy (*n* = 7)	Bell’s palsyBrain tumourSpace occupying lesion(*9 differentials in total*)	732	Bell’s palsyNerve palsy(*5 Read codes in total*)	42	5	1	6
	Bell’s palsyBrain tumourLyme disease(*7 differentials in total*)	721					
5: Recurrent synovitis of knees (*n* = 7)	OsteoarthritisRheumatoid arthritisGout(*9 differentials in total*)	543	Knee painSynovitis(*4 Read codes in total*)	32	7	7	7
6: Multiple EM rashes (*n* = 7)	Insect biteFungal infectionLyme disease(*11 differentials in total*)	633	Non-specific rashErythema migrans(*6 Read codes in total, one GP wouldn’t code the case*)	21	3	6	0
7: Heart rhythm abnormalities (*n* = 7)	ArrhythmiaPalpitations(*11 differentials in total*)	43	Palpitations(*2 Read codes in total, one GP wouldn’t code the case*)	5	6	2	2
8: Fatigue, post-exertional malaise, anxiety, headaches, and memory issues (*n* = 5)	Thyroid problemsChronic fatigue syndromeDepression(*8 differentials in total*)	433	Tired all the time(*3 Read codes in total, one GP wouldn’t code the case*)	3	5	1	3
	Thyroid problemsChronic fatigue syndromeDepressionLyme disease(*7 differentials in total*)	3221					
9: Fatigue, arthralgia, poor ability to concentrate, myalgia, mood swings (*n* = 7)	DepressionAnaemiaThyroid problems(*13 differentials in total*)	533	FatiguePolyarthralgia(*4 Read codes in total, one GP wouldn’t code the case*)	32	7	5	1
10: Non-engorged tick attached to scalp (*n* = 6)	Cutaneous hornForeign bodySkin lesionTick(*10 differentials in total*)	2222	Skin lesionTick bite(*4 Read codes in total*)	22	4	1	0
11: Poor fine motor movements, rash 2 months previously at scout camp. This had been treated with erythromycin (*n* = 8)	DepressionNeurological conditionLyme disease(*18 differentials in total*)	441	Feeling lowLow moodNeurological symptoms(*4 codes in total, one GP wouldn’t code the case*)	222	7	0	3

Thematic analysis of the interview transcripts yielded five main themes relating to coding behaviour. ‘GP personal and professional experience’ described both the GP’s personal experience of the presentation (for example, if they or a family member has had Lyme disease), and their professional experience (for example, whether they have diagnosed and managed a similar case presentation previously). ‘Clinical evidence’ referred to the set of information required by a GP to build a confident clinical diagnosis. This includes: clinical history and presentation of the patient; diagnostic test results; and knowledge of research data and disease guidelines. ‘Diagnostic uncertainty’ was defined by Bhise *et al* as *‘* *the subjective perception of an inability to provide an accurate explanation of the patient’s health problem* *'*.^23^ ‘Professional integrity and defensive practice’ was defined by the GP performing professional activities in an honest, professional, and ethical manner in accordance to professional and practice guidelines; defensive practice referred to the practice of recommending further diagnostic testing or treatment that is not necessarily the best option for a patient, but which protects the GP against potential complaints or litigation. ‘Patient-sourced health information and beliefs’ described the patient’s knowledge, diagnostic information, and values around a certain medical condition. For example, their thoughts about the cause, diagnostics, and value of different treatments for a specific disease.

GP personal and professional experience underpins the other four themes. Owing to this, examples of this core theme will be illustrated with quotations nested among the other themes’ quotations.

### Clinical evidence and diagnostic uncertainty

Without strong clinical evidence, a GP may have diagnostic uncertainty and choose a relatively non-specific Read code:

*‘I think first, "What is the primary complaint?", and unless there is something definitive, I tend to code with the primary complaint or not code at all. So for this patient, what is the primary complaint? Numbness, dropping things, or is it his blue hands?’* (GP5, case 3)

GP5 is unsure of the diagnosis and does not have the prior personal experience or strong clinical evidence to choose acrodermatitis chronica atrophicans as the definitive diagnosis. They, therefore, suggest that they will adopt a highly generic code for this patient.

GP3 showed similar behaviour. They wanted stronger evidence and lacked personal experience, resulting in diagnostic uncertainty. They chose not to code at all.

*‘If I can’t diagnose, I will pick the main symptom to code. I will always do this unless I’m almost a hundred per cent positive of the diagnosis. Sometimes,* *i* *f I’m really not sure, I will write everything in free text and not code anything.’* (GP3, case 2)

GP6 shared the same view succinctly:

‘*If I don’t know what it is, then I won’t code it. Otherwise it gives the appearance of certainty, where there isn’t.’* (GP6, case 6)

The need to build a strong clinical evidence base was strengthened by multiple GPs enquiring whether the patient in case 1 had a significant travel history, and that some would perform internet searches or ask colleagues about unusual rashes. Similarly, in case 10, all GPs asked for a better description about the firmness and texture of the tick.

This shows that GPs need a certain level of supportive evidence, which will vary for each condition and for each GP, to have certainty and confidence in their diagnosis. This can be seen in the results of case 1 (Table 3) where the majority of GPs thought the case was Lyme disease, but subsequently coded the patient with a rash. This suggests that without diagnostic certainty, a non-specific presenting symptom will be chosen as the Read code rather than one with a definitive diagnosis.

This was exemplified by several GPs who noted that they had heightened diagnostic uncertainty regarding Lyme disease, and without sufficient confirmatory evidence, would not code it as such. This may be owing to a lack of personal experience:

*‘Lyme disease is a possibility here. But I wouldn’t leap to it without a history of a tick bite.’* (GP5, case 11)

With more supportive evidence or personal experience, they may have greater confidence in their diagnosis and coding. In many cases, Lyme disease was never discussed. It is probable that either the GPs’ knowledge of the various presentations of Lyme disease was poor, or that the GPs would not consider a differential diagnosis of Lyme disease until they had greater clinical evidence, or that Lyme disease would only enter their list of differential diagnoses through a diagnosis of exclusion.

Personal experience can become its own form of evidence, and when this occurs diagnostic certainty improves and the Read code selected becomes highly relevant:

*‘This is a tick; I’ve been bitten many times before.’* (GP4, case 10)

GP8 had recently been involved in the recent management of a Lyme disease case:

*‘I had a patient diagnosed in the last couple of months; a child with non-specific knee pain. We initially suspected an infected knee joint. He’s now been successfully treated and has been fine since.’* (GP8)

This resulted in this GP having much greater personal awareness of Lyme disease presentations; this GP confidently placed Lyme disease on their differential lists and chose relevant Read codes.

### Professional integrity and defensive practice

Professional integrity drives certain coding behaviour. GP7 showed frustration around how other members of their practice do not respect practice policy nor understand the need for appropriate coding:

*‘We’re told off if we don’t code all patients. Others in the practice use the code "chat with patient", it drives me mad!’* (GP7)

This was seen more acutely where a GP felt hindered in coding Lyme disease owing to both a lack of diagnostic evidence, and the desire to protect themselves and their colleagues from litigation:

*‘I would never write Lyme disease on a patient’s record until I had a positive lab diagnosis. I’m wary because of potential litigation, and I don’t want to cause problems for future doctors treating that patient.’* (GP2)

### Patient-sourced health information and beliefs

The current landscape around Lyme disease and the lack of trust in certain information sources results in diagnostic uncertainty:

*‘I won’t code Lyme disease until they’d seen an NHS specialist. I’d be very suspicious if it* [laboratory results] *was a "high street" or "internet" lab, so I would arrange serology to be sent to a local lab.’* (GP4, case 8)

*‘I would be very suspicious of results from a non-NHS supported lab. She also has no supportive history for Lyme disease.’* (GP3, case 8)

One of the GPs helped place this in context by discussing their own experiences with a cohort of patients in their practice:

*‘We’ve had a few patients, around five, who have formed a little chronic Lyme disease group. All white, middle-aged, very wealthy women. They have spent thousands at xxx* [a private clinic and laboratory offering unvalidated testing and treatment for Lyme disease] *and claim to have Lyme disease, but present more with ME* [myalgic encephalomyelitis].*’* (GP5)

Lyme disease sits within a very confused medical landscape. NICE guidelines^15^ have recently been published and the national incidence is low.^18,24^ GPs should, therefore, have confidence in making a clinical diagnosis. There are now multiple patient groups and charities raising awareness about Lyme disease. However, a lot of sources discuss ‘chronic Lyme disease’, a condition not currently accepted by medical organisations.^15,16,21,25,26^ ‘Chronic Lyme disease’ is the narrative that is often portrayed in the media rather than that of research or medical organisations.^27^ These contrasting narratives make it a challenge for both the GPs and patients to clearly identify scientific fact.^28^ This was made evident by two of the GPs interviewed:

*‘Loads of people* [patients] *are asking about it* [Lyme disease] *in the practice, though I’ve only seen four or five over my career.’* (GP5)

*'There are so few* [Lyme disease] *specialists across the country. A friend of mine has Lyme, so I know the difficulties.’* (GP4)

This complex environment could result in GPs being hesitant to diagnose and code a patient with Lyme disease without confirmation from a laboratory or referral unit.

## Discussion

### Summary

This study has identified key themes describing GPs’ coding behaviour in primary care, utilising the example of Lyme disease diagnosis. Coding behaviour appeared malleable, shaped by clinical evidence, professional integrity and defensive practice, diagnostic uncertainty, patient-sourced health information and beliefs, and personal and professional experience. This has implications for electronic health records database research based on diseases with non-specific presentations, as there is a strong likelihood that some GPs will be reluctant to code such diseases without prior diagnostic confirmation.

From a research perspective, clinical coding of health records may underestimate the true incidence of certain diseases in primary care. This is especially true when traditional methods of validation, for example, diagnostic test results or referral letters, would not be appropriate for these conditions. The masked questionnaire methodology can be used to validate incidence figures based on the Read codes of conditions that, like Lyme disease, have varied and non-specific presentations that may be challenging to diagnose in primary care.

From a clinical perspective, this study highlights that GPs often do not code patients with a specific Read code until they have enough clinical information to move past their working diagnosis onto a definitive diagnosis.^29^ There can be negative consequences associated with the allocation of the wrong diagnostic code. These misdiagnoses can lead to unnecessary treatments and diagnostics (which can have negative health impacts for the patient), unneeded costs of treatment, delayed diagnosis and treatment of the true condition, and potential litigation.^29^ By waiting to code with diagnostic certainty, the GP does not place the patient at unnecessary risk and protects themselves professionally. In many clinical scenarios, by waiting to collect all the relevant clinical information to build a definitive diagnosis, the GP will be benefitting the patient. However, this may not hold true with diseases with pathognomonic presentations, such as the erythema migrans presentation of Lyme disease, where diagnostic testing is not recommended. Delayed diagnosis and treatment could lead to more severe health consequences.

### Strengths and limitations

The results of this study have identified several themes underlying GP coding behaviours in primary care. They highlight a complexity around coding, shaped by both unconscious and conscious drivers. The resulting lack of confidence to code non-specific presentations needs to be accounted for in future research based on primary care EHRs. The applicability of these findings need to be explored with other conditions and with other GPs.

An aim of this study was to explore whether the methodology described could be used to validate Lyme disease Read codes used in disease incidence research. Of 11 cases, only two were given specific Lyme disease codes (Table 3). While it would seem inappropriate to define the accuracy of these codes because of the small sample size, one could speculate that the specificity is likely to be high as Lyme disease codes were only used for ‘textbook’ clinical presentations, resulting in very few false positive codes and a high positive predictive value. Conversely, the sensitivity is likely to be low, with large numbers of more variable cases not being coded. If a GP selects a Lyme disease code, it is highly likely that the patient has Lyme disease. However, many cases are likely to remain uncoded and any incidence figures derived from these codes are likely to underestimate the true incidence of presentations to primary care. By masking GPs to the clinical context, a new methodology is provided that can be utilised to validate incidence figures of other Read codes.

The limitations of this work include selection bias and the small sample size. The GPs were chosen for convenience and were unlikely to be representative of the national GP population. The practices were in areas with a low laboratory-confirmed incidence of Lyme disease,^18^ but much of England has a low incidence, with only southern central England having a relatively higher incidence. Such GPs may reasonably not recognise what is a rare disease in their location, possibly explaining some of their reluctance to code. If this study had been performed at a national scale, these biases could have been examined and explored in more depth, providing greater insight into coding behaviours.

### Comparison with existing literature

The data suggest that when a GP is presented with a case of vague symptomology or they are unsure of the diagnosis, they will often not code with a highly specific and definitive code such as ‘Lyme disease’, instead coding the patient’s primary complaint, the main symptom, or not coding at all. They will only code with a definitive diagnosis code if the clinical evidence or personal experience is strong enough to justify it. In a video-based study of GPs, 60.9% of ‘skin’ presentations, 50.0% of ‘neurological’ presentations, and 83.5% of ‘general and unspecified’ presentations would not have a Read code recorded.^30^ These categories are reflective of some of the clinical presentations of Lyme disease. It may be no surprise that so few GPs selected ‘Lyme disease’ as a Read code. In the video-based study, only 36.7% of clinical presentations resulted in a Read code being used.^30^ This work supports the present study's conclusions and highlights a large amount of underreporting in EHRs, and not just in non-specific clinical presentations.

Themes identified here match previous research about coding behaviour.^4–7^ These include diagnostic uncertainty and a GP’s personal and professional experience. In these studies, GPs state that they were fearful of the potential negative impact of giving a patient a definitive diagnosis through a Read code. This fear may also explain the reduced likelihood of giving a specific diagnosis; it was not explicitly stated by the GPs in the authors' research. Previous research has identified that ‘emotional motives’ can drive decision-making behaviour.^6^ This can be based simply on the recognition of a similar case or on the previous negative experiences of an incorrect or missed diagnosis of a patient. These works and this present study highlight that human emotion and experience may be a larger driving force, in some instances, of decision-making behaviour than the medical evidence alone.

Themes identified previously but not here include: the lack of direct patient benefit; targets and financial incentives driving coding; and the negative impact coding can have while trying to lead a patient-centred consultation.^4^ These topics may not have been discussed here as no questions were directly asked about coding behaviour. This may indicate that, for this set of GPs, these themes were not as important as the ones identified. This research builds on and supports the evidence that diagnostic uncertainty and personal experience are critical elements of a GP's coding behaviour. The other three themes of clinical evidence, professional integrity and defensive practice, and patient-sourced health information and beliefs, need to be explored to assess whether they can be generalised across the UK primary care clinician population.

### Implications for research and practice

This study explored a masked methodology that could be utilised in future research to validate incidence figures calculated through Read codes for conditions that cannot be validated through traditional methods. The widespread dissemination of masked questionnaires to GPs (for example, via the internet), where they are questioned about how they would code a variety of clinical presentations, could give an idea of the validity or under or overrepresentation of Read codes associated with that disease. This methodology should be explored further nationally, for a variety of different conditions, to understand how robust and practical it is to perform. If successful, it could help open new avenues of primary care EHRs research that currently remain poorly explored.

GPs, who are using Read code-based research studies to inform their clinical practice, need to be aware that incidence figures may not reflect what they see in practice and are likely to underestimate disease. In addition, any surveillance system based on specific Read codes is likely to underreport cases. GPs should treat these research designs to appropriate scrutiny and interpretation.

There is a reluctance by GPs to code with specific diagnostic Read codes when they are presented with a patient with vague or unfamiliar symptomology. The masked questionnaire methodology offers a new approach to validate incidence figures calculated from Read codes of non-specific conditions. The reluctance to code poses many problems for research based on primary care EHRs and further research is needed to understand what drives GPs’ coding behaviour.
